# Wide Swath Stereo Mapping from Gaofen-1 Wide-Field-View (WFV) Images Using Calibration

**DOI:** 10.3390/s18030739

**Published:** 2018-03-01

**Authors:** Shoubin Chen, Jingbin Liu, Wenchao Huang, Ruizhi Chen

**Affiliations:** 1 State Key Laboratory of Information Engineering in Surveying, Mapping and Remote Sensing, Wuhan University, Wuhan 430079, China; shoubin.chen@whu.edu.cn (S.C.); hwc0632@whu.edu.cn (W.H.); ruizhi.chen@whu.edu.cn (R.C.); 2 Collaborative Innovation Center of Geospatial Technology, Wuhan University, Wuhan 430079, China; 3 The Department of Remote Sensing and Photogrammetry and the Center of Excellence in Laser Scanning Research, Finnish Geospatial Research Institute, 02430 Masala, Finland

**Keywords:** Gaofen-1, stereo mapping, satellite image, wide-field-view, wide swath

## Abstract

The development of Earth observation systems has changed the nature of survey and mapping products, as well as the methods for updating maps. Among optical satellite mapping methods, the multiline array stereo and agile stereo modes are the most common methods for acquiring stereo images. However, differences in temporal resolution and spatial coverage limit their application. In terms of this issue, our study takes advantage of the wide spatial coverage and high revisit frequencies of wide swath images and aims at verifying the feasibility of stereo mapping with the wide swath stereo mode and reaching a reliable stereo accuracy level using calibration. In contrast with classic stereo modes, the wide swath stereo mode is characterized by both a wide spatial coverage and high-temporal resolution and is capable of obtaining a wide range of stereo images over a short period. In this study, Gaofen-1 (GF-1) wide-field-view (WFV) images, with total imaging widths of 800 km, multispectral resolutions of 16 m and revisit periods of four days, are used for wide swath stereo mapping. To acquire a high-accuracy digital surface model (DSM), the nonlinear system distortion in the GF-1 WFV images is detected and compensated for in advance. The elevation accuracy of the wide swath stereo mode of the GF-1 WFV images can be improved from 103 m to 30 m for a DSM with proper calibration, meeting the demands for 1:250,000 scale mapping and rapid topographic map updates and showing improved efficacy for satellite imaging.

## 1. Introduction

By the end of the twentieth century, a series of major breakthroughs had been made in the fields of space technology and information technology, resulting in significant changes to the fields of surveying and mapping. The development of Earth observation systems continues to change the nature of survey and mapping products as well as the methods for updating maps. Thus, satellite images have become another important source of information in addition to aerial photogrammetry. Among the optical satellite mapping methods, the multiline array stereo mode and agile stereo mode are undoubtedly two of the most common methods for acquiring stereo images.

**Figure 1:**
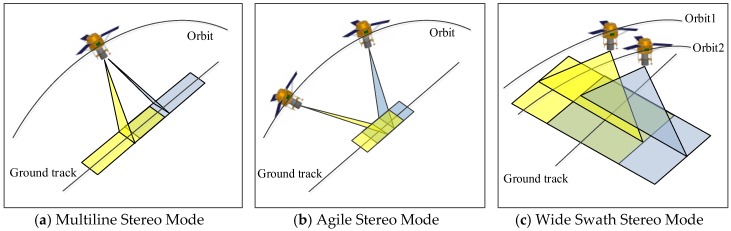
Three different stereo mapping mode. (**a**) Multiline array cameras attain multiple images at different angles, baselines, and overlapping areas, (**b**) one camera observes the same area from different angles at the same orbit by the attitude maneuver, (**c**) one camera observes the same area from different orbits without requiring attitude agility.

As shown in Figure 1a, the multiline array stereo mode uses multiline array cameras to image the surface and acquire multiple images at different angles, baselines, and overlapping areas. Because this method acquires strip images along a track, it is capable of surveying and mapping a wide area. The SPOT-5 HRS camera [1,2,3,4] and the Terra ASTER camera [5,6] use the two-line array stereo mode, whereas the Ziyuan-3 triple linear-array camera [7,8] and the MappingSatellite-1 camera [9,10,11] adopt the three-line array stereo mode. However, due to the narrow width (generally less than 50 km) of the multiline array, the revisit period may be up to two or three months, which results in a low temporal resolution. In short, the multiline array stereo mode has a wide spatial coverage and a low temporal resolution.

As shown in Figure 1b, the agile stereo mode uses one camera to observe the same area at different angles and forms a stereo image pair to obtain stereo information. This mode is typically used to acquire two or more times the number of observations of the same area at different angles using the attitude maneuver of the satellite pitch or roll axis. Relying on high satellite agility, the agile stereo mode can make rapid stereo observations of an area (generally in a few seconds along a track or a few hours across a track). IKONOS [12], GeoEye [13], QuickBird-2 [14], WorldView [15], SPOT-6 and 7 [16], and Pleiades [17,18,19] use this stereo mode for surveying and mapping. However, due to satellite agility, the agile stereo mode cannot easily acquire a complete strip of stereo images covering a broad area and can only focus on one area, such as an urban area. In short, the agile stereo mode has a high-temporal resolution and a narrow spatial coverage.

Thus, the conflict between the temporal resolution and spatial coverages of these two modes limits many remote sensing applications, such as rapid updates of medium scale topographic maps, global change detect, etc., which often require wide spatial coverages and high-temporal resolution. In June 2009 and October 2011, the Advanced Spaceborne Thermal Emission and Reflection Radiometer (ASTER) provided two versions of the Global Digital Elevation Model (GDEM). Although the ASTER GDEM achieves a global 30-m resolution, meeting the demand of a 1:250,000 scale topographic map, the data have a poor temporal resolution and make it difficult to rapidly update maps and detect global change.

In this study, we show how the wide field and short revisit period of images in the wide swath stereo mode can address this issue. This mode is typically used to acquire two or more times the number of observations of the same area from different orbits. As shown in Figure 1c, the wide swath stereo mode uses one camera to observe the same area from different orbits and forms a stereo image pair using the WFV of the camera without requiring attitude agility. Compared with classic stereo modes, the wide swath stereo mode relies on a wide swath (e.g., 800 km) and can rapidly obtain numerous stereo observations of a certain area (generally within a few days) as well as provide a wide coverage for survey and mapping purposes. In short, the wide swath stereo mode has both a wide spatial coverage and high-temporal resolution, which can meet the demands for rapidly updating maps and detecting global changes.

At present, while high-resolution wide swath images are less common because of the limitations of satellite camera hardware, the Gaofen-1 (GF-1) wide-field-view (WFV) images, with their total swath width of 800 km, multispectral resolution of 16 m and revisit period of four days [20,21], are used to implement the wide swath stereo mode. In addition, calibration of the lens to correct for the radial distortions is used in generation of digital surface models (DSMs) from SPOT-5 [22], and there are nonlinear system errors in GF-1 WFV images. Therefore, calibration is necessary and vital in the computation of GF-1 3D stereo models for more accurate DSM. In this paper, we first present the key processes behind the wide swath stereo mode with calibration. Then, we perform GF-1 WFV experiments to demonstrate DSM accuracy improvement after calibration and the validity of the wide swath stereo mode, which is our research purpose. Finally, we present a discussion and concluding remarks.

## 2. Materials and Methods

### 2.1. Overview of GF-1 WFV

GF-1 is the first satellite of the Chinese high-resolution Earth observation system. The main purpose of GF-1 is to make major technological breakthroughs, such as those in optical remote sensing technology (high-spatial, multispectral, and high-temporal resolutions), multi-image mosaic and fusion technology, high-precision and high-stability attitude control technology, high-reliability low-orbit satellite technology, and high-resolution data processing and application technology [20].

The GF-1 satellite design parameters are shown in Table 1. The satellite has a sun synchronous orbit and is equipped with two high-resolution (HR) cameras and four WFV cameras. The nadir resolution of the HR panchromatic camera is 2 m, and that of the HR multispectral camera is 8 m. The total swath of the HR cameras is 60 km, and thus, the revisit period is typically 41 days. The nadir resolution of the WFV camera is 16 m over a total swath of 800 km, and this camera has a revisit period of 4 days.

**Figure 2:**
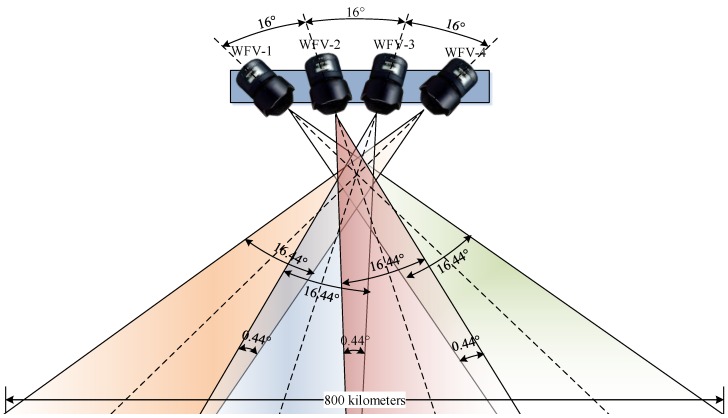
Field design of the GF-1 WFV cameras. The field of view (FOV) of single camera is 16.44°, and the overlap FOV between adjacent cameras is 0.44°.

In this study, we use the WFV cameras. The field design of the GF-1 WFV cameras is shown in Figure 2. The field of view (FOV) of the camera is 16.44°, and the overlap FOV between adjacent cameras is 0.44°. The angle between the center sights of WFV-1 and WFV-4 is up to 48°. By taking the wide swath characteristics into account, it is possible to apply WFV-1 and WFV-4 to stereo mapping.

However, because the primary goals of the GF-1 WFV camera are for use in land and resource surveys, the nonlinear system errors of the image, especially the distortion error, are less of a consideration in the camera design and data processing. The nonlinear system error of the images will seriously influence the stereo mapping, so a calibration should be applied to the WFV camera to acquire non-distorted images in advance. Then, an analysis of the intersection accuracies between WFV-1 and WFV-4 should be performed to demonstrate the feasibility of the image acquisition. Finally, the processing procedure for the wide swath stereo mapping using GF-1 WFV images must be specified.

### 2.2. Calibration

To acquire a high-accuracy digital surface model (DSM), the nonlinear system distortion in the GF-1 WFV images should be detected and compensated for in advance. Traditional calibration methods usually require a high-accuracy geometric calibration field (GCF) that covers the entire image across the satellite path to acquire sufficient ground control points (GCPs) [17,23,24]. However, due to the wide swath size of the GF-1 WFV images, it is difficult to obtain enough GCPs from the GCF to cover all rows in one GF-1 WFV image, especially when considering the high construction costs and site constraints of the GCF.

Huang et al. [25] propose a multicalibration image method to solve the GF-1 WFV image calibration problem. In this method, the calibration images are collected at different times, and their different rows are covered by the GCF. Then, the GCPs covering all the rows can be obtained and can be used with the modified calibration model to detect distortion. Experiments show that this method can increase the GF-1 WFV image orientation accuracy from several pixels to 1 pixel, thereby eliminating nearly all the nonlinear distortion. In this study, we use this method to detect and correct the GF-1 WFV-1 and WFV-4 images.

The calibration model for the linear sensor model is established based on [7]:

(1) $$\left[\begin{array}{c} X_{S}\\ Y_{S}\\ Z_{S} \end{array}\right]=\left[\begin{array}{c} X\left(t\right)\\ Y\left(t\right)\\ Z\left(t\right) \end{array}\right]+m\cdot R\left(t\right)\cdot R_{U}\left(t\right)\cdot \left[\begin{array}{c} x+\Delta x\\ y+\Delta y\\ 1 \end{array}\right]$$

where [X(t), Y(t), Z(t)] indicates the satellite position with respect to the geocentric Cartesian coordinate system, and R(t) is the rotation matrix converting the body coordinate system to the geocentric Cartesian coordinate system. Both these parameters are functions of time and are, therefore, functions of scan lines. Here, [x+Δx,  y+Δy,  1] represents the ray direction when the *z*-coordinate is a constant with a value of 1 in the body coordinate system. Furthermore, *m* denotes the unknown scaling factor, and $\left[X_{S},\;Y_{S},\;Z_{S}\right]$ is the ground position of the pixel in the geocentric Cartesian coordinate system. *R_U_* is the offset matrix that compensates for the exterior errors, and (Δx, Δy) denotes the interior distortion of the image space.

*R_U_* can be expanded by introducing additional variables [26,27,28]:

(2) $$R_{U}\left(t\right)=\left[\begin{array}{ccc} \cos\varphi & 0 & \sin\varphi \\ 0 & 1 & 0\\ -\sin\varphi & 0 & \cos\varphi \end{array}\right]\cdot \;\left[\begin{array}{ccc} 1 & 0 & 0\\ 0 & \cos\omega & -\sin\omega \\ 0 & \sin\omega & \cos\omega \end{array}\right]\cdot \;\left[\begin{array}{ccc} \cos\kappa & -\sin\kappa & 0\\ \sin\kappa & \cos\kappa & 0\\ 0 & 0 & 1 \end{array}\right]$$

where ω, φ and κ are rotations about the *X*, *Y*, and *Z* axes of the body coordinates, respectively, and should be detected to eliminate exterior errors. Note that images collected at different times have different exterior errors, and thus, the number of *R_U_* values correspond to the number of images.

As mentioned above, multicalibration images are collected at different times and have different exterior errors (the installation errors may be the same) but the same interior error. The strong correlation between the exterior and the interior errors will inevitably influence the interior error in different calibration images. The interior error in the image space varies with the calibration images and is difficult to fit using the classical 5 order polynomial model [25].

The additional parameters $c_{j},\;d_{j},e_{j},f_{j}$ are introduced, and the modified polynomial model can be written as [25]

(3) $$\left\{ \begin{array}{l} \Delta x=a_{0}+a_{1}s+a_{2}s^{2}+\cdots +a_{i}s^{i}+c_{j}+d_{j}s,\\ \Delta y=b_{0}+b_{1}s+b_{2}s^{2}+\cdots +b_{i}s^{i}+e_{j}+f_{j}s, \end{array}\right.\begin{array}{c} 0\leq i\leq 5\\ 2\leq j\leq n \end{array}\;\;\;$$

where the variables $a_{0},\;a_{1},\cdots ,a_{i}$, and $b_{0},\;b_{1},\cdots ,b_{i}$ describe the distortion; *s* is the image coordinate across a track; *n* represents the number of calibration images; and $c_{j},\;d_{j},e_{j},f_{j}$ represent the modified parameters of each calibration image (except for the base image). Note that the images collected at different times have the same distortions.

Based on Equations (1)–(3), the functional relationship of the image point and parameters can be derived as Equation (4) in a simple style:

(4) $$\begin{array}{c} x=x\left(a_{0},\;\cdots ,\;a_{i},\;b_{0},\;\cdots ,\;b_{i},\;\omega ,\;\varphi ,\;\kappa ,\;c_{j},\;d_{j},e_{j},f_{j}\right)\;,\\ y=y\left(a_{0},\;\cdots ,\;a_{i},\;b_{0},\;\cdots ,\;b_{i},\;\omega ,\;\varphi ,\;\kappa ,\;c_{j},\;d_{j},e_{j},f_{j}\right)\;, \end{array}\;\begin{array}{c} 0\leq i\leq 5\\ 2\leq j\leq n \end{array}$$

Equation (4) is the basic calibration model of the proposed method.

By taking partial derivative and linearization for Equation (4), the error equation can be written simply as:

(5) V=At−L

where $t=\left[da_{0},\;\cdots ,da_{i},db_{0},\;\cdots ,db_{i},dc_{2},\;dd_{2},de_{2},df_{2},\cdots ,dc_{n},\;dd_{n},de_{n},df_{n},d\omega_{1},d\varphi_{1},d\kappa_{1},\cdots ,d\omega_{n},d\varphi_{n},d\kappa_{n}\right]$ represents the correction to the calibration parameters of images. *A* is coefficient matrix of the error equation, and *L* is the constant vector. Equation (5) is the basic error equation of the proposed method in the paper.

The corresponding normal equation of Equation (5):

(6) $$A^{T}At=A^{T}L$$

The correction to the calibration parameters *t* from the normal Equation (6) will be:

(7) $$t={\left(A^{T}A\right)}^{-1}A^{T}L$$

After the correction to the calibration parameters *t* is calculated, the calibration parameters can be updated.

### 2.3. 3D Stereo Model and Analysis

**Figure 3:**
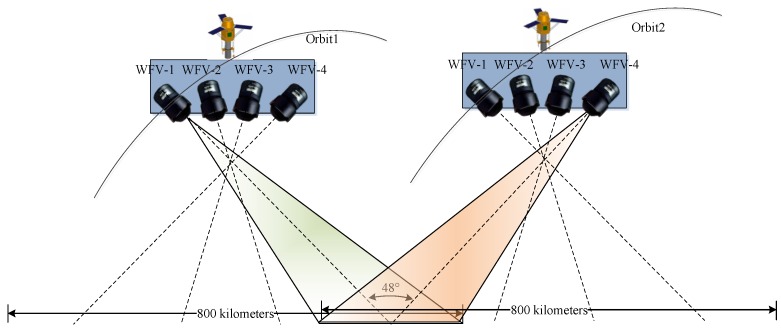
Forward intersection method of the stereo partners. Partners are made up of the GF-1 WFV-1 and WFV-4 images from different orbits with common coverage. The angle between the center sights of WFV-1 and WFV-4 is up to 48°.

The stereo partners for 3D stereo model are made up of the GF-1 WFV-1 and WFV-4 images from different orbits with common coverage (Figure 3). Corresponding points acquired by the semi-global matching (SGM) method [29] enable to reconstruct the 3D location of the object point on the terrain. Forward intersection is done via iterative least squares adjustment using 2*n* (for *n* stereo partners) observation equations [30,31]. Normally, in this research on Gaofen-1, *n* is 2 and 4 equations are established per stereo tie point for the derivation of the 3 object space coordinates including planimetry and elevation. The initial values for the object space coordinates are derived from an affine transformation using the corner coordinates given by the image provider. Initial height values are taken from the mean height of the area under investigation. Normally, convergence is achieved after several iterations.

According to [32,33], the ratio R between the vertical accuracy and horizontal accuracy is written as follows:

(8) $$R=\frac{h_{error}}{v_{error}}=\frac{H}{S}$$

where *h_error_* represents the horizontal error and *v_error_* represents the vertical error. *S* is the baseline length, and *H* is flight height. Thus, the horizontal error can be calculated as follows:

(9) $$h_{error}=\frac{H}{S}\cdot v_{error}$$

The flight height of GF-1 is 644.5 km, while the baseline between WFV-1 and WFV-4 is approximately 600 km. The calibration accuracy *e_c_* is approximately 1 pixel, and the corresponding point matching accuracy *e_m_* is approximately 0.5 pixels. Because the nadir resolution *res_nad_* is approximately 16 m, the resolutions of WFV-1 and WFV-4 (*res*) are determined by Equation (10) considering the swing angle *θ* (half of the angle between the two camera center sights):

(10) $$res=\frac{res_{nad}}{\cos^{2}\theta }$$

Thus, the vertical and horizontal errors for WFV-1 and WFV-4 are as follows:

(11) $$\begin{array}{ll} v_{error} & =\left(e_{c}+e_{m}\right)\cdot res=\left(e_{c}+e_{m}\right)\cdot \frac{res_{nad}}{\cos^{2}\theta }\\ & =\left(1+0.5\right)\times \frac{16}{\cos^{2}\left(24\right)}=28.8\;(m)\\ h_{error} & =\frac{H}{S}\cdot \left(e_{c}+e_{m}\right)\cdot v_{error}=\frac{644.5}{600}\times 28.8=30.9\;(m) \end{array}.$$

According to the stereo analysis, the planimetric and height accuracies for the GF-1 WFV-1 and WFV-4 cameras corresponded to approximately 29 m and 31 m. As the calibration and matching accuracies are approximate values, the stereo accuracy is merely a reference value that differs slightly from the actual value at each pixel.

### 2.4. Processing Procedure

**Figure 4:**
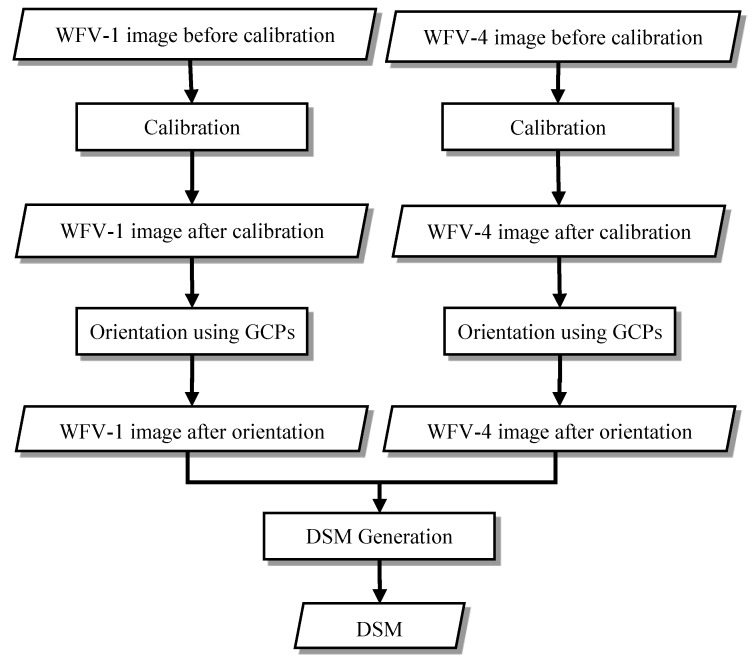
Wide swath stereo mapping process using the GF-1 WFV images. Step 1: the calibration is to correct for the systematic nonlinear distortion and acquire post-calibration images. Step 2: to determine elements of absolute orientation in two images. ‘GCPs’ stands for ground control points. Step 3: to acquire the corresponding points and generate a digital surface model (DSM) from point cloud.

The process for wide swath stereo mapping using GF-1 WFV images is shown in Figure 4. There are three main processes: calibration, orientation using GCPs, and DSM generation. Of these, the calibration and orientation using GCPs are applied to raw WFV-1 and WFV-4 images, respectively, whereas the DSM generation uses the WFV-1 and WFV-4 images after orientation.

First, the calibration method in [25] is used to detect and correct for the systematic nonlinear distortion error and to acquire post-calibration images. Note that distortion detection is performed only once for each camera during the calibration process, and the calibration parameters can be used continuously by compensating the images.

Then, the orientation using the GCPs is applied based on the affine model, which is the most common orientation model, resulting in post-orientation images. This process should be performed on each image because the orientation without the GCPs differs for each image. The orientation process eliminates most of the random errors in the images.

Finally, the calibration parameters for camera distortion and exterior orientation parameters from the affine model are sent to DSM generation. The SGM method [29] is introduced to acquire the corresponding points and point cloud, and then the DSM is generated from the point cloud. Note that the WFV-1 and WFV-4 images should have some overlap when using this method.

## 3. Experiments

### 3.1. Datasets

#### 3.1.1. Calibration Images

To acquire the calibration parameters, we collected some calibration images. Detailed information on the WFV-1 and WFV-4 calibration images is listed in Table 2 and Table 3, respectively. The GCPs are acquired via the method introduced in [25] using the GCF, and the sample range represents the GCF coverage of the start and end rows of the images across the track.

#### 3.1.2. Stereo Mapping Images

**Figure 5:**
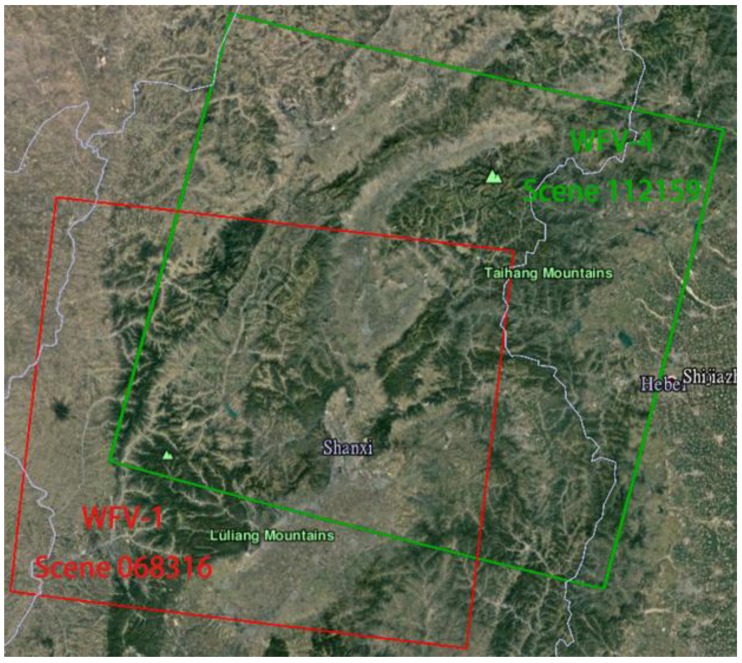
Coverage of the stereo mapping images.

Scene 068316 (WFV-1) and scene 112159 (WFV-4), covering the Shanxi province in China, were collected as stereo mapping images. Detailed information on the stereo mapping images is listed in Table 4, and the spatial coverages of the stereo mapping images are shown in Figure 5. Figure 5 shows that the overlap across the tracks of the stereo mapping images is up to 60%.

### 3.2. Geometry Calibration

The calibration parameters are calculated according to Huang et al. [25]. In [25], the residual errors before and after the compensation for the distortions of the calibration images using the GCPs from the GCF demonstrate that all the distortions have been corrected and the calibration parameters are effective for the calibration images.

After calculating the calibration parameters via the proposed method, it is important to verify whether the calibration parameters can be used in other validation images. Considering the goal to validate the effect of calibration parameters for compensating camera distortion, the affine model for images based on four GCPs was adopted as the exterior orientation model, removing other errors caused by exterior elements [34,35].

Because the GCF has a range restriction and because the swath width of the GF1 WFV camera reaches 200 km, the check points (CPs) from the GCF can only cover some rows of each image. Thus, the exterior orientation will absorb some interior errors and influence the orientation accuracy of the whole image. Considering the resolution of the GF1 WFV (16 m) and the horizontal positioning accuracy of Google Earth (less than three meters) [36,37], it is proper and feasible to manually extract corner points or feature points from Google Earth as CPs to evaluate the orientation accuracies and illustrate the influence of compensation.

As shown in Table 5, the maximum orientation errors without the calibration parameters are approximately 5.5 pixels in the WVF-1 camera and approximately 11 pixels in the WVF-4 camera. The orientation accuracies without the calibration parameters are only two pixels in the WVF-1 camera and approximately five pixels in the WVF-4 camera. These errors are partly the result of the distortion in the original scenes. Thus, when the original scenes are compensated with the calibration parameters acquired by the proposed method, the maximum orientation errors are reduced to less than two pixels for both cameras. The orientation accuracy level after the calibration consistently exceeds one pixel, especially the accuracies of scenes 125567, 061400, 112159 are reduced to approximately 0.5 pixels, illustrating that the proposed method can provide effective distortion compensation for the WFV-1 and WFV-4 cameras.

### 3.3. Orientation Accuracy of Stereo Images

The orientation errors of the stereo scenes 068316 and 125567 are shown in Table 5. Before calibration, the maximum error is up to 2.7 pixels for scene 068316 (WFV-1) and 9.2 pixels for scene 112159 (WFV-4). The root mean square (RMS) error is up to 1.4 pixels for scene 068316 (WFV-1) and 5.2 pixels for scene 112159 (WFV-4). After calibration, the maximum error is up to 1.0 pixels for scene 068316 (WFV-1) and 1.1 pixels for scene 112159 (WFV-4). The RMS error is up to 0.6 pixels for scene 068316 (WFV-1) and 0.5 pixels for scene 112159 (WFV-4).

**Figure 6:**
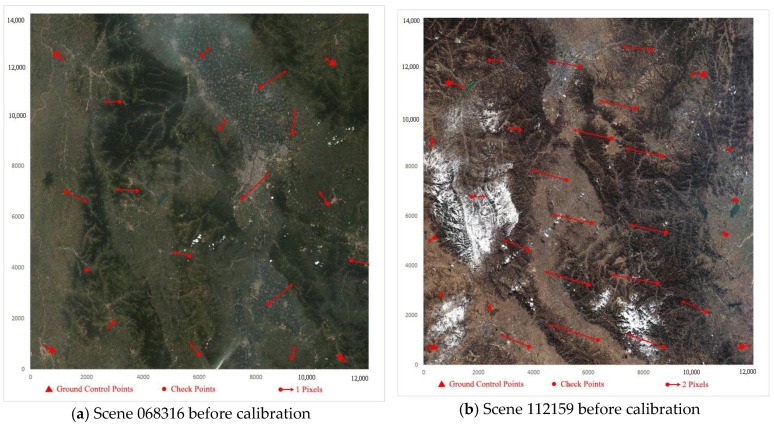

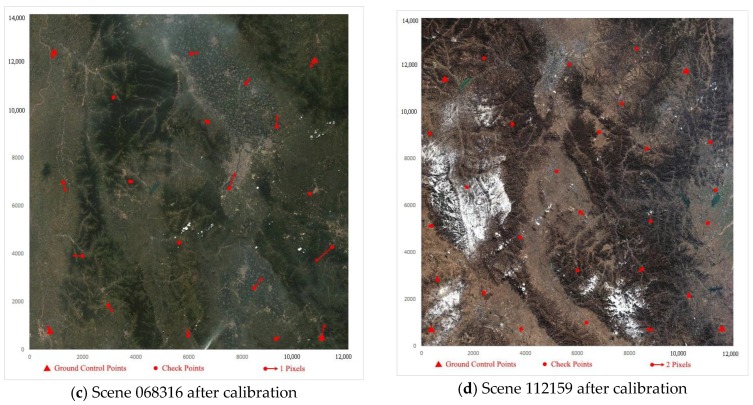
Orientation errors for the stereo mapping images. The red triangles in four corners stand for four ground control points (GCPs) for the affine model (the exterior orientation model). The red points stand for check points (CPs) for orientation accuracy assessment. The longer the red arrow, the more the orientation residual. In (**a**,**b**), the four corners are more accurate than the middle region. In (**c**,**d**), the residual errors become less and random due to that the calibration eliminates the nonlinear system error.

In addition, the orientation residual plots before and after the calibration of scenes 068316 and 112159 are shown in Figure 6. Before calibration, as shown in Figure 6a,b, the plots show that the four corners are more accurate than the other regions because the affine model with four GCPs cannot completely absorb the higher-order distortion effects, especially in the middle region. After calibration, as shown in Figure 6c,d, it can be seen that the accuracy level is consistently approximately one pixel, and the residual errors are random. In short, the nonlinear system error has been eliminated after the calibration, and the images are undistorted images whose residual system errors can be absorbed by the affine model with four GCPs.

Thus, the orientation accuracy has been improved after calibration, and the results after orientation can be used to generate the DSM.

### 3.4. Stereo Mapping

#### 3.4.1. Digital Surface Model (DSM) Generation

The calibration parameters for compensating camera distortion and exterior orientation parameters from the affine model based on four GCPs are used in DSM generation. To compare the accuracies before and after calibration, the SGM method is used on the stereo scenes 068316 and 112159 to generate a large number of corresponding points. Then, the corresponding points are intersected via forward intersection to generate a point cloud. Finally, the point cloud is directly transformed into a DSM with no filtering.

**Figure 7:**
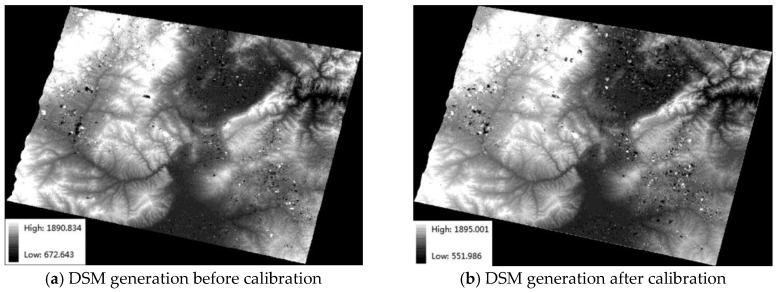
DSM generation.

Figure 7a,b show the DSM generation results before and after calibration, respectively. Although there are a few incorrect results due to poor radiation quality, most areas obtain a complete terrain. Thus, it is possible to use wide swath images in stereo mapping. In other words, the DSM generation results in Figure 7 verify the feasibility of stereo mapping with the wide swath stereo mode.

#### 3.4.2. Elevation Accuracy Validation

**Figure 8:**
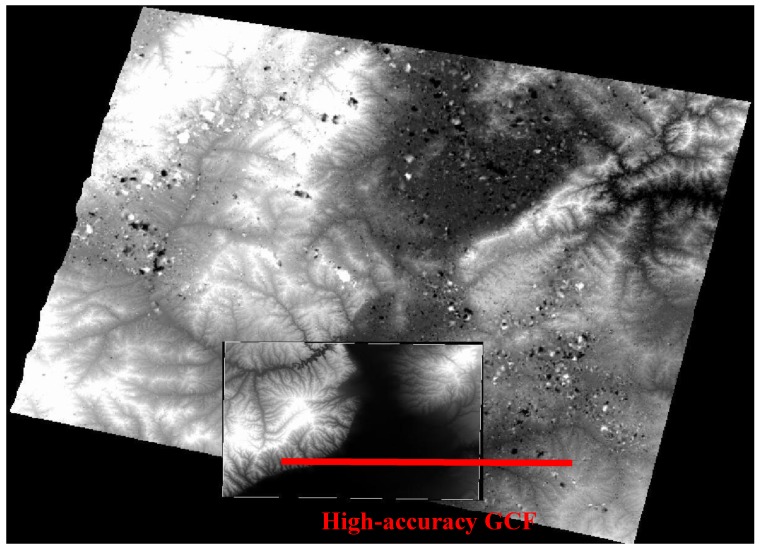
Geometric calibration field (GCF) spatial coverage.

To validate the elevation accuracy, we introduce a high-accuracy GCF whose horizontal and elevation accuracies are 1.6 m and 1.5 m, respectively. The coverage of the GCF is shown in Figure 8. Due to the wide swath of GF-1 WFV, the GCF covers only part of the DSM area. We adopt two analysis methods to verify the accuracy: a profile analysis along the red line in Figure 8, and a global analysis to calculate all elevation errors.

**Figure 9:**
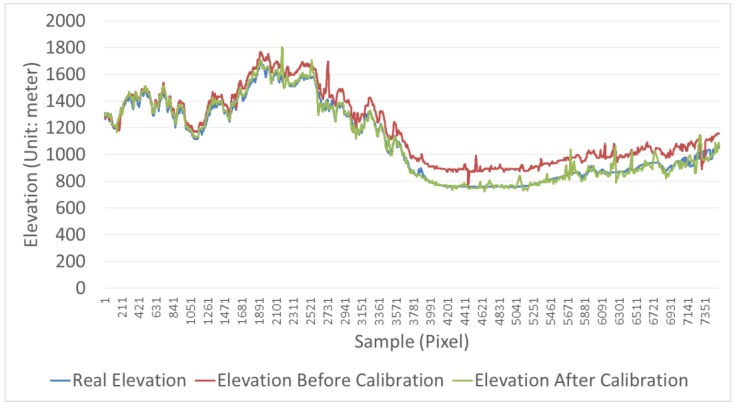
Elevation profile plot.

**Figure 10:**
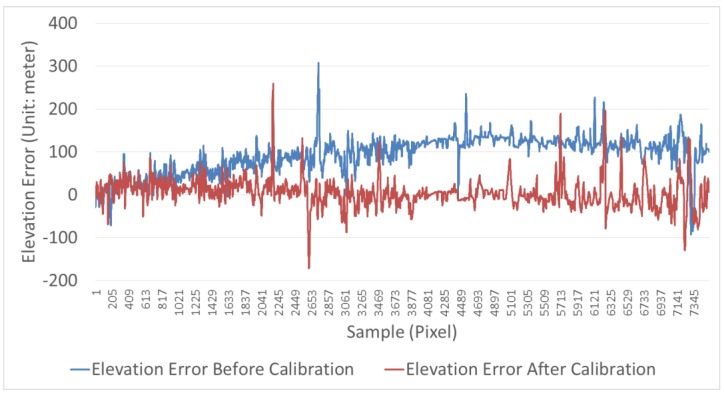
Elevation error before and after calibration.

Figure 9 is the elevation profile plot along the red line, containing the real elevation, the elevation before calibration, and the elevation after calibration. The elevation after calibration closely resembles the real elevation, while the difference between the elevation before calibration and the real elevation gradually increases with the growth of pixel sample number. This phenomenon is more obvious from a perspective of the elevation error before and after the calibration compared with the real elevation in Figure 9. The figure shows that the elevation before calibration is within a few meters of the real elevation. However, the greater the number of pixels, the greater the difference between the elevation before calibration and the real elevation, by as much as a hundred meters. The reason of the phenomenon in Figure 9 and Figure 10 is that the nonlinear system error in the image has been eliminated after calibration. In short, Figure 9 and Figure 10 by a profile analysis demonstrate DSM accuracy improvement after calibration and that the calibration results in a relatively good DSM.

**Figure 11:**
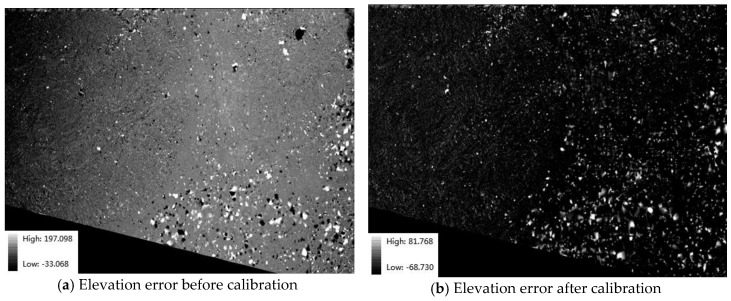
Elevation error with the high-accuracy GCF. (**a**) A systematic error in the DSM before calibration, (**b**) the error is random in the DSM after calibration.

**Figure 12:**
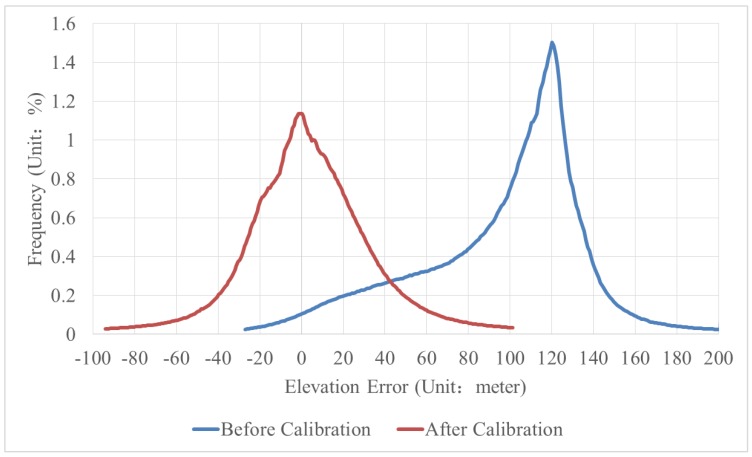
Statistical plot of the elevation error. The peaks are at 120 m and 0 m respectively, before and after calibration. Statistical plot tends to a more standard normal distribution after calibration.

Figure 11 and Figure 12 and Table 6 are the results of a global analysis to calculate all elevation errors. Figure 11 shows the elevation error compared with the high-accuracy GCF. The figures show that there is a systematic error in the DSM before calibration, whereas the elevation error is random in the DSM after calibration. The contrast demonstrates that calibration detects and compensates the nonlinear system error in the image, resulting in DSM accuracy improvement.

The corresponding statistical plot of the elevation error is shown in Figure 12, and the elevation error statistics are shown in Table 6. Before calibration, there is one peak (120 m) in the statistical plot, which results in a mean elevation error of 95.927 m and an RMS error of 103.850 m. The systematic deviation phenomenon fits the increasing trend gradually with elevation error. In addition, the plot shows a biased normal distribution. After calibration, there is only one peak at zero meters. Thus, the mean elevation error is approximately 4.107 m, and the RMS error is 30.116 m, leading to a more standard normal distribution of the plot. In general, the global statistical analysis in Figure 12 and Table 6 further indicates that calibration brings an obvious drop of elevation error or a significant improvement of DSM accuracy.

In addition, the 30 m elevation accuracy is consistent with the stereo analysis result (31 m), meeting the demand of the 1:250,000 scale mapping and rapid updates of the topographic map. The low elevation accuracy is the result of the low resolution and poor radiometric quality, as opposed to the wide swath stereo mapping mode. Considering the 16 m nadir resolution and poor radiation quality, the elevation accuracy is significantly improved after calibration. 

## 4. Conclusions

This paper proposes a wide swath stereo mode method that is characterized by both a wide spatial coverage and a high-temporal resolution. Compared with classical stereo modes, the wide swath stereo mode is capable of obtaining a wider range of stereo images over a short time period. The GF-1 WFV images with a total swath of 800 km, a multispectral resolution of 16 m and a revisit period of four days, are used in experiments. Nonlinear system errors in GF-1 WFV images is detected and compensated for in advance, and calibration bring a significant improvement of DSM accuracy. The results show that the wide swath stereo mode of the GF-1 WFV images can reach an elevation accuracy of 30 m for a DSM at proper calibration conditions, which meets the demand of the 1:250,000 scale mapping and rapid updates of the topographic map, and demonstrates the feasibility and efficacy of this mode for satellite imaging. 

Moreover, given the limited nadir resolution of 16 m and poor radiation quality of the GF-1 WFV images, the 30 m elevation accuracy is still relatively low, although the elevation accuracy is significantly improved after calibration. We suggest that by using higher resolution wide swath images of improved radiation qualities, the wide swath stereo mapping mode will deliver better results with the proper calibration.

## Figures and Tables

**Table 1:** Gaofen-1 satellite design parameter table.

Item	Area	Technology Performance
Orbit	Orbit Type	Sun Synchronous Circle Orbit
	Average Height	644.5 km
	Descending Time	10:30 a.m.
	Regression Period	41 days
	Revisit Characteristic	No Swing: WFV Camera 4 days; HR Camera 41 days
		Swing: HR Camera 4 days
High-Resolution (HR) Camera (PAN/MS)	Nadir Resolution	Pan < 2 m; MS < 8 m
	Swath	≈60 km
	Field of View	≈6.4°
Wide-Field-View (WFV) Camera	Nadir Resolution	<16 m
	Swath	>800 km
	Pixel Size	0.0065 mm
	Focal Length	270 mm
	Field of View	>48°
Attitude Control	Control Mode	Three Axis Stability, Ground Orientation
	Pointing Accuracy	<0.1° (three axes, 3σ)
	Stability	<5 × 10^−4^°/s (three axes, 3σ)
	Maneuverability	Swing: ±25°

**Table 2:** Details of the GF-1 WFV-1 calibration images.

Scene ID	Camera	Area	Imaging Data	Number of GCPs	Sample Range (Pixel)
068316	WFV-1	Shanxi	10 August 2013	15,800	6300–9000
108244	WFV-1	Shanxi	7 November 2013	18,057	10,200–12,000
125565	WFV-1	Shanxi	27 November 2013	19,459	3200–5700
126740	WFV-1	Shanxi	5 December 2013	14,551	500–2700

**Table 3:** Details of the GF-1 WFV-4 calibration images.

Scene ID	Camera	Area	Imaging Data	Number of GCPs	Sample Range (Pixel)
061400	WFV-4	Shanxi	30 July 2013	2754	0–1800
101766	WFV-4	Shanxi	23 October 2013	13,099	4700–7000
108857	WFV-4	Shanxi	8 November 2013	12,791	6000–8500
113764	WFV-4	Shanxi	20 November 2013	16,361	2000–4500
120856	WFV-4	Henan	28 November 2013	1410	8500–11,000

**Table 4:** Details of the stereo mapping images.

Scene ID	Camera	Area	Swing Angle (°)	Imaging Data
068316	WFV-1	Shanxi	27.611	10 August 2013
112159	WFV-4	Shanxi	−27.611	16 August 2013

**Table 5:** Orientation accuracy before and after calibration for validation images (units: pixels).

Camera	Scene ID	Number of CPs	Status	Line	Sample	Max.	Min.	RMS
WVF-1	068316	20	Ori.	0.916	1.069	2.692	0.207	1.410
			Cla.	0.430	0.437	0.991	0.130	0.613
	079476	28	Ori.	0.840	1.921	5.538	0.512	2.097
			Cal.	0.646	0.635	1.788	0.088	0.906
	125567	26	Ori.	0.966	1.721	3.173	0.541	1.973
			Cal.	0.384	0.433	1.072	0.079	0.579
	132279	26	Ori.	0.790	1.991	4.922	0.249	2.142
			Cal.	0.525	0.505	1.198	0.054	0.728
WVF-4	061400	30	Ori.	1.879	5.551	11.121	0.084	5.861
			Cal.	0.369	0.453	1.188	0.040	0.584
	112159	26	Ori.	1.552	4.942	9.252	0.222	5.180
			Cal.	0.422	0.393	1.118	0.084	0.577

Ori.: original, Cal.: calibration.

**Table 6:** Elevation error statistics before and after calibration (units: meter). The elevation accuracy can improve from 103 m to 30 m.

Item	Min.	Max.	Mean	STD	RMS
Before Calibration	−27.098	201.809	95.927	39.786	103.850
After Calibration	−94.076	101.343	4.107	29.835	30.116

STD: standard deviation, RMS: root mean square.
